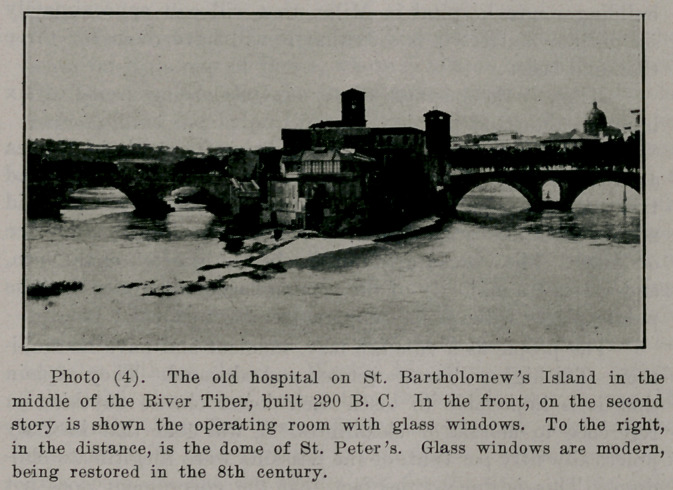# A Trip through Europe and the Hospitals

**Published:** 1914-11

**Authors:** L. C. Fischer

**Affiliations:** Atlanta, Ga.


### Journal-Record of Medicine

Successor to Atlanta Medical and Surgical Journal, Established 1855
and Southern Medical Record, Established 1870

**OWNED BY THE ATLANTA MEDICAL JOURNAL COMPANY**

PUBLISHED MONTHLY

*Official Organ Fulton County Medical Society, State Examining Board, Presbyterian Hospital, Atlanta, Birmingham and Atlantic Railroad Surgeons' Association, Chattahoochee Valley Medical and Surgical Association, Etc.*

EDGAR BALLENGER, M. D., Editor
BERNARD WOLFF, M. D., Supervising Editor
A. W. STIRLING, M. D., C. M., D. P. H.; J. S. HURT, B. Ph., M.D.
GEO. M. NILES, M. D., W. J. LOVE, M. D., (Ala.); Associate Editors
R. R. DALY, M. D., Associate Editor
E. W. ALLEN, Business Manager

**COLLABORATORS**

W. F. WESTMORELAND, M. D., General Surgery
F. W. McRAE, M. D., Abdominal Surgery
H. F. HARRIS, M. D., Pathology and Bacteriology
E. B. BLOCK, M. D., Diseases of the Nervous System
MICHAEL HOKE, M. D., Orthopedic Surgery
CYRUS W. STRICKLER, M. D., Legal Medicine and Medical Legislation
E. C. DAVIS, A. B., M. D., Obstetrics
E. G. JONES, A. B., M. D., Gynecology
R. T. DORSEY, JR., B. S., M. D., Medicine
L. M. GAINES, A. B., M. D., Internal Medicine
GEO. C. MIZELL, M. D., Diseases of the Stomach and Intestines
L. B. CLARKE, M. D., Pediatrics
EDGAR PAULLIN, M. D., Opsonic Medicine
THEODORE TOEPEL, M. D., Mechano Therapy
A. W. STIRLING, M. D., Etc., Diseases of the Eye, Ear, Nose and Throat
BERNARD WOLFF, M. D., Diseases of the Skin
E. G. BALLENGER, M. D., Diseases of the Genito-Urinary Organs

VOL. LXI. ATLANTA, GA., NOVEMBER, 1914. NO. 8

### A TRIP THROUGH EUROPE AND THE HOSPITALS.

BY DR. L. C. FISCHER, ATLANTA, GA.

(Continued.)

By the time we were ready to leave Rome we had begun to study carefully the tipping evil that has been so much discussed. Once or twice we had been held up by some hotel attaches, porters, bell boys, etc.; but not until we reached the depot to take the train for Florence were we so forcibly impressed with the systematic way in which this is carried out. Our baggage was handled by a portor, who, after getting it into a compartment, came for his tips. He charged 6 cents for each heavy suit case and 4 cents for each light suit case or hand grip. When he was settled with, he carried the amount to a man who stood guard at the gate and was apparently in charge of all porters. He had a large iron box suspended from his neck in which there was a slot for the reception of all moneys. It was explained to us that each porter when he received a tip carried it and placed it in this box; that at the expiration of the day the box was opened by some official of the railroad or depot; that they took out a certain per cent of the amount received and divided the balance among the various porters, and that they were not paid any salaries, except what they made from handling baggage. It was readily seen that if a man were allowed to keep all of his tips he would be making more than an ordinary living wage.

I investigated very carefully the best way to handle the tipping part of our trip; found it to be a general custom to give practically 10% of your bills for services; that is, if staying in a hotel for five days at thirty francs a day, which would amount to $30.00, the most satisfactory way to do was to pay the head waiter 10% of this amount, or $3.00, which was then divided by him among the servants. A peculiar thing to me was that no bills were paid in the hotel office; but the head waiter invariably brought it to you the morning before leaving. There is one man in the hotels throughout Europe known as the portier, who is the highest official from his viewpoint that you come in contact with. He is the one who furnishes all the information, and, if you attempt to divide the tips, expects the greatest amount. It is amazing to see the amount of local information that he has and the work he does, it being his duty to assign guests to their rooms, to see that all baggage is properly cared for, to make and settle all bills, and to be the chairman of the general reception committee. While as a rule they impress you as being more or less ignorant of the general topics of the world, at the same time they speak from three to ten languages fluently.

Leaving Rome, we went by rail to Florence. The railroad travel in Italy is divided into four classes, first, second, third and fourth. The only difference between first and second class is that the upholstery in the first class is red, while that in the second class is grey. This may sound the least exaggerated, but, upon very close inspection, this was the only difference perceptible. In the third class the seats are not upholstered and the sanitary conditions are almost unbearable. It is barely possible that some of the cars may have been cleaned out within

Photo (1) is an ambulance conveying a sick man from the poorer district to the hospital, this owned by an insurance company. A great many others of the same design are owned by charitable organizations.

the last year. If this be so, there is no evidence. I am persuaded to believe that the Italians' idea of cleanliness is carried all the way from their attention to their bodies and their homes on down even to public conveyances.

The fourth class are cars with cross seats that reminded me very much of the old benches used when I was a school boy. I remember very well that they were slabs of woods taken from the outside of logs with holes bored through them, and pegs driven in to support them or act as legs. The floors of the cars reminded one very much of a pigsty. While the number of people chewing tobacco is nothing to compare with our country, and they do not have the expectorating to contend with, still they eat all kinds of fruit and throw the residue on the floors. Even from these filthy floors I have seen a passenger pick up the ends of cigars and cigarettes and save them to make snuff or smoking tobacco. Every time that I bought a cigar in Europe, the seller asked to clip the end of it, which he explained he was saving for soldiers' smoking tobacco.

The laborers in Italy are very poorly paid. They make an average of sixty cents a day. The railroad people are paid from forty to fifty dollars per month. Street car conductors make from twenty to thirty dollars per month. All of these people, however, are working for the government, that is, the railroads, street cars, and a great many of the public conveyances, such as cabs, hacks, etc., and after a certain length of time they are placed on a pension which varies in amount according to the time that they have been in service.

There are numerous small towns in the mountains along the way from Rome to Florence, which are entirely deserted, this due to the fact that so many Italians are leaving Italy and going to the States or to other lands of health and freedom, and too, where they can make a respectable living and above all things be free to do and think as they please. The whole world knows that Rome is a veritable parasite, not only upon Italy, but the nations of the earth. As soon as we were out of the city we came into the poorly cultivated areas of farm land surrounding this prehistoric remains of former glory upon which are planted numerous fields of alfalfa and small patches of vegetables. Further along was nothing but hay fields and cattle raising, which continued on to the beautiful city of Florence.

At Florence there were no especially interesting medical points. We were shown the beautiful paintings of Raphael, Michael Angelo and others until my head swam, this being really the center of Italian art. We saw copies of some of the beautiful studies in anatomy by Rembrant and other artists;

Rembrant, however, being the most famous painter of anatomical subjects. We were shown the beautiful old bridge known as the "Ponte Vecio," where Dante first met Beatrice. Even though this has been years ago, they show and sell you postcards of the actual persons. All of these things have to be taken "cum granum salis," and even then you feel as if the dose should be doubled.

A little side trip made from Florence was to see the "leaning tower of Pisa." We were there on one of the festive days

Photo (2). An ambulance conveying an injured person from the scene of the riot to the hospital; said to contain the Assistant Chief of Police of Rome; this riot taking place on the second day of our visit in Rome in June. On account of excessive speed (?) made the patient was dead before reaching the hospital, he having to go approximately two miles in this conveyance.

and were not allowed to walk up the 196 steps—178 feet—to the top of this wonder of the world. It is built of marble and leans 13 feet out of true. I smile when the thought of the cartoonist who draws Mr. Diggs is brought to mind—he makes him to go to the leaning side, put his shoulder to the tower and call for help to keep it from falling until something can be done. Was much inclined to take the same action to keep it from falling on the great crowd who were worshipping the bones of some departed saint.

From Florence we went to Venice, reaching there in the afternoon in a heavy rain. When we left the depot and went out to take a car or bus for the hotel, found that they were all really water wagons.

After having read for years and dreamed of beautifully decorated and gaily painted gondolas, handsome and gaudily attired gondoliers, with beautiful and varied colored lights adorning their small crafts, one is depressed by the approach of something that resembles a hearse, all painted black, with trimmings very similar to those on a coffin. As we got into the first one, I felt that I was almost a corpse going to my own funeral, and that the gondolas following were part of the funeral procession—the only thing lacking would be the funeral dirge. As we went into the city, this feeling of gloom deepened. Instead of the delightful water ways which I had dreamed of as being bedecked on either side with elegant homes, beautiful flowers and handsome women showing from the windows, and the strains of grand opera floating out into the air as the gondolier would join his voice with that of the singers, there are narrow and dark water passages, from which the odor was almost unbearable, with numerous dead cats and other animals floating past us; the homes on either side were filthy and delapidated; the only sound to awaken us from our reverie was the occasional signal from the gondolier, as he approached a crossing, which sound was most weird and blood-curdling. Most all of the gondoliers wear earrings and have a voice something similar to a fog horn on a dark night off Hatteras.

We finally reached the hotel, which was the former home of a Doge. In ancient days, when Venice was one of the strongest empires, it had a population of a million and a half. Today it is only the mourning remains of what has been, being now a city of a hundred and twenty-five thousand people, who are afflicted with poverty, sickness and disease.

Built, as it is, on piles, practically every home in the city has front, side or rear entrance on some one or even more waterways, There are a few streets, but, in any case, you have to walk up over a bridge to cross the water passages.

We were shown through the dungeons where the prisoners in other days were placed next to a lead wall and excessive heat turned on until they were burned to death.

Then we were shown the ancient and supposedly horrible "Bridge of Sighs," where prisoners were supposed to go over and never come back. Indeed, if one were confined in one of these dungeons, which are in most cases built of rock, with no possible entrance for air or light, the food passed in through a funnel, it seemed to me that death would be welcome, and that he would hail the day with delight on which he knew he was to be executed. We were also shown where a prisoner was

Photo (3). On the right is the old bridge running from the city of Rome across a portion of the River Tiber to St. Bartholomew's Island. Built 200 years B. C. This is built of Ferro-Concrete.

placed in a dungeon, his hands fastened behind him, and a board fixed under his neck, on which was placed fresh food three times a day. It was impossible for him to get any of this, and anyone helping him would be subject to the same punishment. This process was kept up until the prisoner starved to death.

After going through these horrible dungeons, one can't help but wonder how Byron ever received an inspiration lying in one of these flat on his back for twenty-four hours without food or water, to write the "Childe Harold." You can better understand how Shakespeare received an inspiration to write the "Merchant of Venice," after visiting the Rialto and the various shops and markets which stand today as described by him.

The fish and other markets have such an odor around them that close inspection is impossible. All of the fruits and vegetables are left uncovered, and thousands of flies are constantly swarming over them. When you see conditions as they exist you can't wonder that cholera is an occasional visitor to that ancient city.

The people are all superstitious, and, in a great many cases, very ignorant. The gondolas are all painted black, and the women wear black shawls, this in mourning for the terrible scourge of cholera that existed in Venice some hundred and seventy years ago. It is a rule that the government has established that all these boats must stay black. It gives one an idea of the lack of development in civilization in this country to see this superstitious idea carried out today as though the terrible plague had existed a few years ago.

In the Grand Canal you are shown the house in which Desdamona and Otello lived, though they never existed except in the mind of fiction.

The hospital in Venice is a very old one, having been built about the thirteenth century. It, too, is built on and over the waterways. The narrowest passages surrounding it have their walls covered with moss and are damp for several feet up. This accounts for a great deal of the rheumatism and pneumonia which are largely prevalent.

One day while gliding along in one of the hearses, we were suddenly awakened by the clanking of a bell. Looking around, we saw approaching at an unbelievable speed an emergency ambulance. This was propelled by four gondoliers. Every boat gave them right of way. It looked strange to find a city of this size without any horses or cows. The only fowls were the pigeons that have been fed on St. Mark's Square for some four hundred years. Incidently these are served to you at your hotel, at which time you have no way to know whether they are some of the younger generation, or whether, like things shown you in Italy, have a record.

From Venice we went to Milan. Milan is a beautiful modern city, with a cathedral that has been in process of construction since the thirteenth century. There have been times when the work has been discontinued, but, during the last hundred years, it has been almost continuous. We were told that the building has already cost over forty millions of dollars. It is built of pure white marble and has over two thousand life size marble statues which surmount an equal number of spires—these carvings by some of the most famous sculptors in Italy. The odors in this old church take one back to the many

Photo (4). The old hospital on St. Bartholomew's Island in the middle of the River Tiber, built 290 B. C. In the front, on the second story is shown the operating room with glass windows. To the right, in the distance, is the dome of St. Peter's. Glass windows are modern, being restored in the 8th century.

ancient churches of Rome and to the catacombs. It is strange to say that even in this most magnificent edifice there are no seats whatever.

Went through the old hospital, which was built in the seventeenth century. It is built around a court and is three stories high, is fire proof, and arranged so that all of the rooms face a veranda, which is on the inside of the court and goes all the way around, forming a sun parlor for the entire length of the hospital. It contains two thousand beds. These beds are for the most part small iron ones and are very neatly kept. The halls in the oldest part of the building are dark and narrow. It is practically impossible to get any ventilation. The hospital was built by a Roman Catholic priest and donated to the Italian government.

The ambulances were the most beautiful that we had seen in Italy, more modern, if possible, than the ones that we have. They have absolute right of way, as the ambulance doctors are given the same authority as the police and gendarmes; they, too, carry large red crosses showing front and rear. They are building a new hospital in Milan that will cost approximately six million dollars; it is said that it will have room for three thousand beds.

Milan is the business center of Italy, being a city of six hundred thousand people.

Great many of the men spend their afternoons in the tea and wine shops. I was amazed to see thousands sitting around tables drinking and talking for hours. With all of this, did not see an intoxicated man in Naples, Rome, Venice, Florence or Milan. The women drink as freely and as often as the men, and too vast a majority of the women smoke cigarettes at these public places without any apparent embarrassment.

The people as a rule are lazy, indolent and have no ambition. The higher class are refined and educated. They explain that it is impossible to teach their lower classes anything in reference to morals or cleanliness. I was told that in Italy practically fifty per cent. of the peasants were illegitimate children. This is due to the fact that the government does not allow soldiers or police to marry until the are financially able to care for a wife and children. The things that they seem to raise the biggest crop of in Italy are children, police and soldiers.

It is a fact that Italy is almost bankrupt; at the same time she has an enormous standing army and supports more police and gendarmes than any other foreign nation of the same size.

Her money on the markets of the world is at a greater discount today than that of any nation of the world except Turkey. We were paid as much as twenty cents on the dollar in excess of the value of our money. Especially did this hold true when we presented American gold.

From Milan we went through the Italian lakes and then through the Alps mountains. It was indeed gratifying and restful to leave Italy and to find oneself in the beautiful country of Switzerland. The meals and hotels were much improved. The railroad travel was much superior to anything we had seen up to this time. The cars were clean and they had drinking water on the train, a thing which we had not found throughout Italy.

After a visit to Lucerne and Enterlarken, we reached the beautiful little city of Berne, where is located the distinguished Dr. Theodore Kocher, who is the father of so many valuable surgical ideas and helpful operations. After one has seen him work for days, he is not surprised that he is one of the famous men of the world. His personality is very pleasant, being a medium sized man of about sixty-five years of age, with brown eyes that are very penetrating, with always a pleasant expression unless there is some break in his technique when he is heard from in no mild terms. Even with this age and having devoted his life, as he has, so faithfully and capably to his profession, his hands are still free from any tremor and he operates without glasses. Was at his clinic two days before, I had the pleasure of seeing him work. His son, a man of apparently thirty-five years of age, is operating practically as much as he is. His work too is beautiful. They are both very slow operators, but the results pay for the time. I, the same as most other men who go to Berne, wanted to see Dr. Kocher do his operation for Goiter, also his resection of the rectum without interfering with the bones, together with his many stomach operations, all of which are done without clamps.

His first case was a very large Colloid Goiter in a man. With the patient on an especially built table, head drawn back, the shoulders raised, he was then strapped to the table both hands and feet. Then the draperies were applied, using a special frame to protect the face and head from the field of the operation. The neck was sponged off with alcohol, and then, with a very small camel's hair brush, he painted with iodine his line of incision, that is, made a mark about an eighth of an inch wide around the neck with the brush and not painting over the surface; at the same time, he marked out the tumor with iodine, the upper and lower poles. In all of these cases he used entirely local anesthesia, which was 1% Novocain; added to this was Adrenalin Chloride 1-1000. Using a record syringe this was introduced deep into the tissues and especially into the gland itself. First, he followed along the course of incision into the skin, then into the deeper structures. We were much impressed that with every movement of his hand he accomplished something. In introducing the needle into the deeper structures of the neck before forcing any of the solution into the tissues, he withdraws the piston of the syringe to be certain he is not in one of the larger blood vessels. This little procedure he does every time before the solution is forced in. Waiting about five minutes after the anesthetic is introduced, he makes his own incision around the neck over the prominent tumor, this being the same as described in many textbooks. With the tissues dissected down to the muscles, he clamps them at their insertion and divides so as to give more room. Each little bleeding vessel is caught and ligated as he goes, using silk entirely. When the gland is exposed and the arteries ligated, before any dissecting is done, the patient is asked to say something to see if the recurrent Laryngeal nerves are free. This is certainly a great advantage. Instead of worrying over the possibility of injuring them, and this anxiety not relieved until the patient is awake, he knows at the time, this made possible by local anesthesia. Several times in the days that I watched them operate did he change a clamp or instrument after some intonation from the patient. He removed much more of the gland than had been my custom to do. He explained that we often left too much, especially in the Exophthalmic variety. After removing the gland, the cut muscles were refastened to their insertion and the wound closed, usually without drainage. The skin is closed with silk, using interrupted stitches all the time. For a dressing a very small strip of gauze bandage was placed over the incision, this plastered down with Collodion. When dried, some light dressing was placed over this. The beds were rolled into the operating room; the patient transferred directly from the table to the bed and put in sitting position with a back rest. Every patient left the operating room smiling and very often talking back to the surgeon answering some pleasantry. His next and next and next cases were goiters. Stood from seven-thirty in the morning until two o'clock in the afternoon watching him operate on nothing but goiters. In his operations for very large or Exopthalmic goiters, he often removes only half of the gland and waits for the patient to react before the second half is removed. Often the removal of the second half is delayed for months or even years. In all cases where the heart is seriously affected he does the operation in at least two sittings.

One is impressed with the simplicity of the operation in his hands, the most major cases being handled with the greatest ease and skill. His local anesthesia is easy because the surgeon has direct control over the people. They look up to the surgeon as to one in great authority.

When there were no goiter operations, his work would be varied. Saw him do his resection of the rectum without interferring with the bones. This he always does as a secondary operation to Colostomy. After his wound at the rectum is healed and the new anus is well formed, he closes the artificial anus and reopens the lumen of the gut. Saw him make a woman a new nose using the posterior surface of the olecranon process of the ulna for the bone. It was taken from the ulna and placed under the skin of the anterior surface of the forearm until he was sure it would live; then the skin of the forearm fastened to the nose and all allowed to unite well before the forearm was cut loose. So many of his surgical conditions were the result of Syphilis. His stomach work was most beautifully done. In no cases did he use a clamp. His technique was as good as the best. All street clothes are left off. They use masks, gloves, etc., using rubber gloves with thin cloth ones over them. He explains that it is so much easier to handle tissue with cloth gloves on. All of the assistants wear cloth gloves over the rubber ones, and often no rubber.

For the general anesthetics they use apparently anyone as an anesthetizer. They use A. C. E. and Ether, also chloroform, according to the condition of the patient. They use a great deal of gas and oxygen.

The hospital is an old one of several hundred beds. Part of the buildings are modern and beautifully kept. The sanitary conditions seem strange to us. Adjoining the entrance from the outside to the operating room, or rather to the theater part of it, is a large toilet for the students, as well as the public. In this the floor was covered with urine and general debris. The urinals were built in the side of the wall into the floor and did not have any water to flush them. They were painted over daily with some vile smelling antiseptic that would smell worse than the decaying urine.

In the student body they have a great many women. So many of the students, both men and women, are Russian Jews. The student body as a whole were very unattractive. They looked in many cases like the regular Russian Jew. In case you were close enough to smell his breath you could be sure who he was. The students go down into the operating amphitheater directly from the toilet room above referred to. They are lectured to in German; all the clinics are held in German, so, unless one understands this language, he is at a loss. Even though they speak English, they do not like to. Their best work is explained in German. I found, too, that the German, Austrian or French physicians are shown a preference over English speaking men.

The operating rooms are large and well lighted, with every modern convenience to make the work easy and most satisfactory. Many of the ideas we have and a great many of the instruments used originated in the master mind that is at the head of this institution. He very often shows things that he has done and intimates that others claim priority. The abdominal dressings are the same as described for Goiter, except that in addition to the piece of gauze directly over the incision, strips are plastered down the same as adhesive pieces—that is, across the abdomen. The first dressing is changed after forty-eight hours. For some months now he has been using sterile sweet milk in the abdomen to prevent adhesions. Though having used it for hundreds of times, he is not ready to make a report as to the probable good to be derived from its nse.

The city of Berne is a very old place. It is divided into the old and the new town. From Berne come all the new ideas and changes in the international postoffices. In the old town the houses are built out over the sidewalk, so that in a rain you can walk all over the town and not get wet. This is a city of 40,000 people located in the heart of the Alps, with snow covered peaks looking down upon it from every direction.

When you consider that possibly drinking snow water is the cause of so many goiters, you are not surprised that about every one person in ten either has one or the scar where it has been removed. Was impressed with the great number of young women and girls afflicted, and especially at the number of men and boys with them. One day just as the school was out, I stood on one of the most prominent street corners and watched the school girls go by. It seemed to me that at least one in five had a perceptible goiter and a great many of them very large. Almost as many men were operated on as women, and too the largest ones seen were on men. There was a noticeable absence of the Exopthalmic variety. In many of the operative cases the heart and eyes were not affected. In one small town near Berne, they say that every person has either a goiter now or the scar where it has been. The people do not seem to look upon them as serious, nor do the surgeons seem to class it as the most major operation.

The question as to the cause of goiter is not settled even by Kocher, the father of the operation, neither its treatment, other than surgical. The water, climate and the earth are supposed to be very important factors in the development of the strumous growths. A great many doctors from all over the world go to Berne to see Dr. Kocher and the great number of goiters, and to attend the greatest clinic of its kind in the world.

25 East Linden Avenue.

(To Be Continued.)

## Figures and Tables

**Photo (1) f1:**
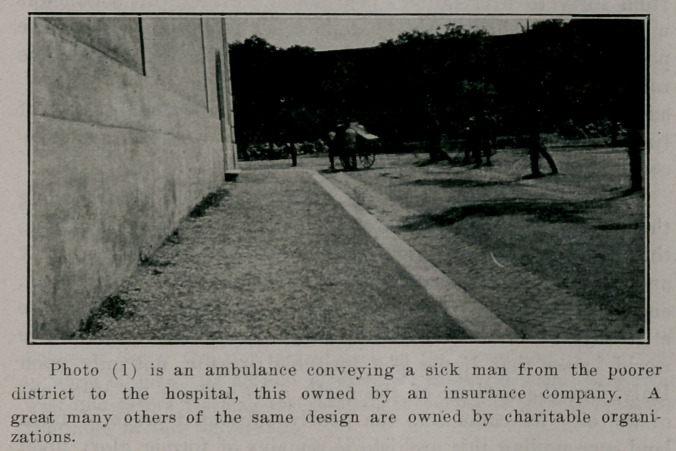


**Photo (2). f2:**
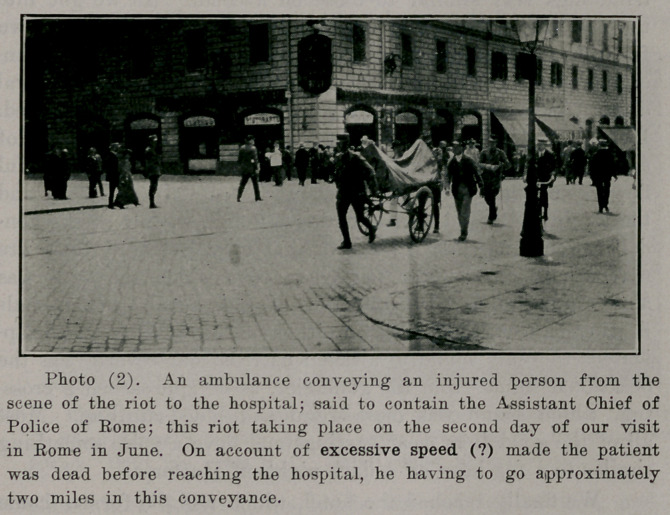


**Photo (3). f3:**
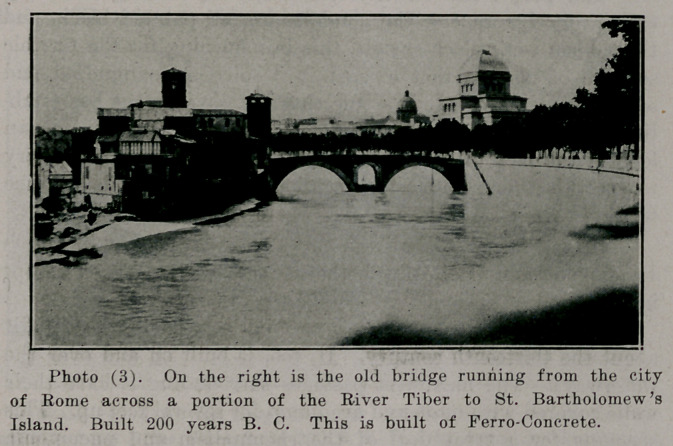


**Photo (4). f4:**